# HEALTHCARE PROFESSIONALS’ PERSPECTIVES ON THE CHALLENGES IN THE VACCINATION OF UKRAINIAN CHILD MIGRANTS AND WAR REFUGEES: FINDINGS FROM A QUALITATIVE STUDY IN POLAND

**DOI:** 10.13075/ijomeh.1896.02478

**Published:** 2024

**Authors:** Katarzyna Lewtak, Aneta Nitsch-Osuch, Anna Dzielska, Tomasz Maciejewski, Anastasiia Atif (Nurzynska), Agnieszka SochoŃ-Latuszek, Katarzyna KukuŁa, Dorota Wiktoria Kleszczewska

**Affiliations:** 1 Medical University of Warsaw, Department of Social Medicine and Public Health, Warsaw, Poland; 2 Institute of Mother and Child, Department of Child and Adolescent Health, Warsaw, Poland; 3 Institute of Mother and Child, Clinic of Obstetrics and Gynaecology, Warsaw, Poland; 4 UNICEF Refugee Response Office in Poland, Warsaw, Poland; 5 Institute of Mother and Child Foundation, Warsaw, Poland

**Keywords:** immunization, refugees, healthcare professionals, Ukraine, vaccination services, social behavioral change

## Abstract

**Objectives:**

The aim of this qualitative research study was to explore the barriers encountered by Ukrainian war migrants and refugees in accessing vaccinations in Poland and the challenges related to delivering vaccinations observed by healthcare professionals (HCPs).

**Material and Methods:**

The study was based on an analysis of data from 18 in-depth interviews with HCPs working with Ukrainian refugees conducted in Poland in July and August 2023. The authors analyzed the data using the UNICEF Journey to Health and Immunization (JHI) framework in order to identify bottlenecks and possible interventions that could solve existing problems in preventive healthcare for migrants.

**Results:**

This qualitative study revealed that at each stage of the JHI, there were challenges related to vaccinating Ukrainian children in Poland, which were similar to those experienced in other countries − gaps in routine immunizations and the need to fill these gaps by ensuring refugee populations are fully included in routine immunization in the host country. The work environment, training, and communication with the Ukrainian mothers contribute to HCPs’ engagement in increasing vaccine uptake among their patients. The HCPs’ attitudes, skills, and experiences impacted their interactions with patients and participation in the immunization process. Healthcare professionals observed that the mother’s journey was influenced by vaccine literacy level, competing priorities, individual barriers of access (e.g., language barrier, costs), as well as feelings associated with the decision to vaccinate a child, including worries about vaccine safety. The surrounding cultural norms, social support, and past experiences with the Ukrainian health system also influenced decisions on vaccinations.

**Conclusions:**

Overcoming barriers related to vaccinations requires a comprehensive approach, starting with expanding HCPs’ knowledge about migrants’ rights to health services, including vaccinations, improving communication between patients and HCPs, building vaccine literacy/trust in vaccinations, and achieving vaccination coverage through tailored and flexible systemic solutions.

## INTRODUCTION

The health of refugees and migrants is important from a human rights, public health, and socioeconomic development perspective, as well as for achieving of the Sustainable Development Goals (SDGs) [1]. In many countries around the world, including Poland, disparities in vaccine-preventable disease (VPD) burden and immunization coverage have been observed between migrants and war refugees and their host populations [2–6].

The findings of the World Health Organization’s Immunization Agenda 2030 show how it is necessary to strengthen national vaccination policies and programs to better address the needs of migrants and war refugees, particularly by improving access to catch-up vaccines across the life course [7]. In light of the UNICEF Immunization Roadmap to 2030, which presents that immunization is one of the world’s most effective public health interventions, averting 2–3 million child deaths every year, public health organizations are making efforts and implementing interventions aimed at increasing vaccination coverage among migrant and war refugee children in host countries [8–14].

Both migrants and war refugees can be vulnerable to developing certain infectious diseases, partly due to the prevalence of these diseases in their country of origin, the hardships of migration, and limited access to health care in the destination country [2,10,15].

Since the populations of migrants and war refugees are socially vulnerable and often face barriers to accessing healthcare services, they may be marginalized or even overlooked in disease prevention and health promotion efforts [4,16].

Therefore, it is crucial to provide this group with the same level of care in terms of prevention and control of infectious diseases, including VPDs, as the care offered to the host population of the receiving country [3,10,12].

The Act of 5 December 2008 on preventing and combating infections and infectious diseases in humans sets forth that individuals staying in the territory of the Republic of Poland for >3 months are required to undergo preventive vaccinations under the Polish national immunization schedule for a given year [17,18].

All Ukrainian children <19 years who arrived in Poland after February 24, 2022, as a result of the outbreak of war in Ukraine are eligible to receive a series of publicly funded vaccinations under the Polish national immunization schedule. The Ministry of Health has published guidelines on the vaccination of children arriving in Poland from war-affected Ukraine. These guidelines prioritize routine vaccinations listed in the National Immunization Program, particularly vaccinations against MMR (measles, mumps, rubella), DTP (diphtheria, tetanus, pertussis), polio, HepB (hepatitis B), and COVID-19 [19].

Despite the availability of publicly funded vaccines, Ukrainian migrant and refugee children in Poland still show suboptimal vaccination coverage and enrollment in immunization registers.

Two years after the outbreak of war in Ukraine, it is currently unknown whether migrant and refugee children residing in Poland or those newly arriving adhere to catch-up immunization schedules and have received all age-appropriate vaccinations required in Poland.

Older migrant and refugee children are less likely to be enrolled in immunization registers and receive vaccinations [4,20].

There are significant barriers to improving vaccination coverage among migrant and refugee children, including low priority for vaccinations among parents, low levels of vaccine literacy, vaccine misinformation spread through the Internet and social media platforms, and other challenges both on the patient side (e.g., financial difficulties) and within the healthcare system (e.g., lack of health provider recommendations) [3,16,21–23].

Healthcare workers are crucial in increasing vaccination rates among migrants, which can also contribute to policy formulation, garner support for policy initiatives, and address vaccination hesitancy [24].

One of the UNICEF aims is to reduce the spread of misinformation and ensure that families have access to accurate data on immunization so they can make the best decisions for their children and to support communities in designing, delivering, and assessing vaccination efforts − starting by listening [8]. The UNICEF Refugee Response Office in Poland has engaged the Institute Mother and Child Foundation to lead the “Say yes to vaccination” initiative, an evidence-based educational project aimed to increase vaccination rates against measles and polio among Ukrainian refugee children in Poland. The project encompassed multiple elements − educational and promotional activities, as well as scientific research that involved qualitative and quantitative methods and focused on Ukrainian mothers and Polish healthcare workers. The objective of this research was to understand the barriers and challenges that Ukrainian refugees face when deciding whether to vaccinate their children in Poland. This paper analyzes the results from the qualitative study conducted among healthcare workers. Another example is the Access to Vaccination for Newly Arrived Migrants (AcToVax4NAM) project, implemented by the National Institute of Public Health − National Research Institute (NIH-NRI) in Poland [13,14].

The aim of this study was to show healthcare professionals (HCPs) observations on vaccinating Ukrainian migrants and war refugees in Poland. These included the challenges faced by doctors and nurses in delivering vaccinations, as well as identification of potential barriers that Ukrainian mothers face in accessing vaccinations for their children.

## MATERIAL AND METHODS

### Study design and population

This qualitative study was conducted to analyze the experiences of 18 HCPs involved in vaccinating Ukrainian child migrants and war refugees in Poland.

Eighteen HCPs working in various types of facilities (primary healthcare [PHC] centers, hospitals) in 3 cities (Warsaw, Kielce, and Piotrków Trybunalski) and 1 town (Poddębice) in Poland were interviewed. These were nurses and physicians from public healthcare facilities such as PHC, maternity and neonatal hospital wards, and private health centers offering services under subscription or fee-for-service models (Table 1).

**Table 1.** Characteristics and demographics of 18 healthcare professionals working with Ukrainian refugees participating in the study, Poland, July and August 2023

Participant	Gender	Role	Place of work	Location
IDI_1	woman	nurse	private PHC center	Warsaw
IDI_2	woman	physician (pediatrician)	public PHC center/hospital	Warsaw
IDI_3	woman	physician (general practitioner)	public PHC center	Kielce
IDI_4	man	physician (pediatrician)	public PHC center	Kielce
IDI_5	woman	nurse	public PHC center	Kielce
IDI_6	woman	physician (general practitioner)	public PHC center	Kielce
IDI_7	woman	physician (general practitioner)	public PHC center	Piotrków Trybunalski
IDI_8	woman	physician (pediatrician)	public PHC center/hospital	Warsaw
IDI_9	woman	nurse	public PHC center/hospital	Warsaw
IDI_10	woman	physician (general practitioner)	public PHC center	Warsaw
IDI_11	woman	physician (pediatrician)	public PHC center	Podd^bice
IDI_12	woman	nurse	public PHC center	Warsaw
IDI_13	woman	nurse	public PHC center	Piotrków Trybunalski
IDI_14	woman	nurse	private PHC center	Warsaw
IDI_15	woman	nurse	public PHC center	Kielce
IDI_16	woman	physician (gynecologist-obstetrician)	hospital	Warsaw
IDI_17	woman	physician (gynecologist-obstetrician)	hospital	Warsaw
IDI_18	woman	physician (pediatrician)	private PHC center/hospital	Warsaw

PHC – primary healthcare.

Kielce – mid-size city 100 000–500 000 inhabitants; Piotrków Trybunalski – small city 50 000–100 000 inhabitants; Poddębice – small town <10 000 inhabitants;

Warsaw – major city >500 000 inhabitants.

Participant inclusion criteria comprised having a lead role in administering childhood vaccinations and experience vaccinating children with migrant and refugee backgrounds. The interviews were based on a prepared interview script. They were conducted in 2023, July 27 − August 28, via Microsoft Teams and lasted approx. 1 h each.

The interviews included discussions on the process of how Ukrainian migrants and war refugees received vaccinations, as well as the challenges faced by migrants and HCPs using the Journey to Health and Immunization (JHI) framework [25].

The JHI framework identifies 6 key steps in the vaccination process:

– knowledge, awareness, and beliefs;
– intent;
– preparation, cost, and effort;
– point of service;
– experience of care;
– after service.

Factors at the individual, community, societal, and political levels impact vaccine access, confidence, and demand at each of these stages.

The data were collected by a specialized polling company. A semi-structured interview schedule was constructed through expert consultations (research experts and clinical providers) and a literature review. To ensure the interview questions were appropriate, they were tested in July 2023 by 2 pilot participants (1 doctor and 1 nurse).

The interviews were recorded, transcribed, and manually coded. To ensure the reliability of coding and interpretation, all transcripts were analyzed by 1 academic researcher and independently by 2 other researchers. Any discrepancies were resolved through dialogue until a consensus was reached.

**Figure 1.**
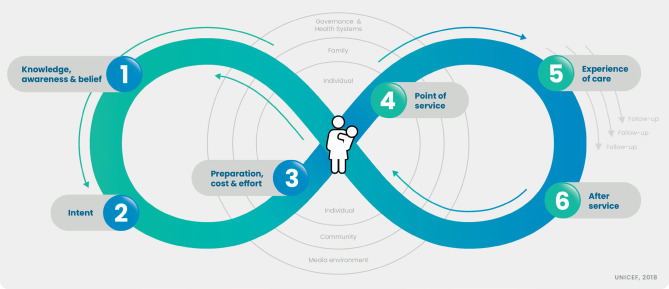
Journey to health and immunization framework

The highlighted subjects corresponded to each of the 6 stages of JHI framework (Figure 1) and enabled an understanding of the experiences and challenges faced by mothers and HCPs before, during, and after vaccination. They were compared with barriers to vaccination and vaccination challenges described in the literature to ensure the reliability and trustworthiness of the data.

Based on Journey to Health and Immunization: Identifying Community Centric Health Interventions [25].

### Ethical considerations and approval

The study’s protocol was approved by the Bioethics Committee at the Institute of Mother and Child (Opinion of the Bioethics Committee at the Mother and Child Institute No. 33/2023 dated April 28, 2023, along with subsequent extensions under Resolution No. 9/2024 dated February 23, 2024) and guided by the ethical principles of the Declaration of Helsinki.

Healthcare professionals identifiable or personal data were not audio-recorded or transcribed to ensure participants’ anonymity. All participants were fully informed about the study, and verbal consent was obtained. They could request to withdraw from participation at any time.

## RESULTS

This study aimed to identify barriers and context-tailored solutions observed by HCPs to increase vaccine uptake and address gaps in vaccinations among Ukrainian child migrants and war refugees.

The concept of JHI invited HCPs to participate in the study to walk in the shoes of the Ukrainian migrant and war refugees population in their journey to vaccination, recognizing their unique experiences. Barriers and facilitators to routine children vaccination for this specific population were identified.

Participant quotes were used to support themes, and participant identifiers were anonymized to protect the participants’ identities (Table 1).

### Knowledge awareness and belief

The sudden influx of refugees was a new situation for the Polish healthcare workers, and they sometimes found it challenging to navigate the new challenges associated with providing care. “I remember the first wave that came. Suddenly, 50 people arrived at our health center […]. There were just five of us women, and we really managed to control the crowd. We stood there, not knowing whether to run away because of the war. It was happening for real, and we had to deal with it […], things we had never encountered before. We had to figure out how to manage” (IDI_1, woman [W], nurse [N]). “The immunization program in Ukraine has been disrupted by the war. We have to reckon with the fact that young children who are now coming to Poland may not have had any vaccinations” (IDI_9, W, N).

According to HCPs, patients from Ukraine initially did not know how to use the Polish healthcare system. “Initially, patients came to the doctor through the hospital emergency department. Later, they started to visit health centers. It was difficult for them to find their way in our country. They didn’t know where to go; everything was new and challenging. But now they already know what to do” (IDI_2, W, medical doctor [MD]). “It was very difficult; mothers were scared by the situation that happened. They had to find themselves in another country, live here and seek help” (IDI_14, W, N).

Mothers’ knowledge did not fully translate into children’s participation in vaccinations. “They are aware that vaccination is necessary. They know that there are good vaccines in Poland. They know that some vaccinations, which are not included in Ukrainian vaccination schedule, are available for free in Poland” (IDI_1, W, N). “I think they want to vaccinate their children. It’s just difficult for us to reach them” (IDI_6, W, MD). “When the war started, it was the peak of the infectious season. In the hospital, we met Polish children of anti-vaccination parents once a month, whereas regarding the Ukrainian children, this happened twice a week. Statistically, these children were more often unvaccinated” (IDI_8, W, MD).

Attitudes towards vaccination in Ukrainian mothers often changed for the better once information about the disease and vaccine safety was given. “It is important how you approach it from a factual standpoint, present the benefits of vaccination, explain the epidemiological situation in Poland, show how some things have changed since the majority of population got vaccinated, and say what it was like before vaccines had been introduced” (IDI_11, W, MD).

“The disease itself may not be frightening, but there is a lack of awareness about the possible complications” (IDI_3, W, MD).

Healthcare professionals believe that misinformation on vaccines spreading through social media or word of mouth has a strong influence on both individual and community vaccine confidence. “They still believe that MMR causes autism, and COVID-19 vaccination causes health problems” (IDI_3, W, MD).

Ukrainian mothers who were unfamiliar with the concept of preventive visits and systematic health screenings, saw no reason to attend medical appointments when there was no health problem. “If they come to medical visits, it’s when they are sick; they rarely come for vaccinations” (IDI_13, W, N). “Sometimes they have been in Poland for two years but have never visited a doctor in Poland. It’s because either the child is not sick, or they have a Ukrainian doctor they can call, or they have brought antibiotics from Ukraine and treat themselves. Sometimes they only come with the child for urgent help. They don’t visit a pediatrician, and they have no family doctor of their own” (IDI_17, W, MD).

Healthcare professionals working with migrants often felt that communication barriers restricted them in informing refugee and migrant patients about vaccines. Communication is important. “To increase vaccination rates, education, information and improved language accessibility are necessary. There is a need to establish access points for Ukrainian patients, where a doctor can explain the mandatory vaccinations in Poland and the differences in vaccination schedules between Poland and Ukraine” (IDI_9, W, N).

Participants emphasized the role of their own knowledge in supporting vaccinations in the following areas: understanding the needs and rights of refugees related to health care; knowledge of the culture, language, and experiences of Ukrainian citizens; knowledge and awareness of services supporting refugees; and practical knowledge in creating individual vaccination schedules for Ukrainian children. “Since 2014, we have already had a large group of Ukrainian patients. This was not our first experience with this group of patients” (IDI_10, W, MD).

Most HCPs stated that they have not received any formal training for working with Ukrainian migrants and war refugees or the necessary materials. There were feelings among participants of the study that they were not adequately trained in refugee-specific health, including catch-up immunization. “As staff, we often sat down, searched in Google, and asked Ukrainian citizens speaking Polish about the vaccinations they had had, in order to check the composition of the vaccines, because we are not familiar with the vaccines used in the former Eastern Bloc. We know which vaccines are given to children in the European Union, but not in Ukraine. It’s a challenge for us” (IDI_9, W, N).

Many participants described how they acquired knowledge through their prior experiences. “Since we joined the EU, we have been aware of the discrepancies between vaccination schedules” (IDI_5, W, N). “I have previously cared for refugees from Chechnya, Kazakhstan, and the Roma” (IDI_11, W, MD).

Participants also described documented information sources. “We have vaccination guides that we rely on. There’s also a Facebook group about vaccinations. There, doctors can consult each other there if they have doubts. Two heads are better than one. I consult cases with colleagues at the health center. We try to help each other. There are also webinars for doctors” (IDI_3, W, MD).

They expressed a strong desire to increase their expertise and confidence in managing the immunization process among migrant and war refugees.

### Intent

Health decisions in general, and vaccination decisions in particular, can be significantly influenced by patients’ trust in the healthcare system and healthcare workers. “We need to talk to them so they know we want to help them... It helps a lot when they see a Ukrainian [a doctor from Ukraine]” (IDI_1, W, N). “When we talk about vaccinations, we plant a seed. Ultimately, they make the decision themselves, but it is me who starts the conversation, and encourages them to read about vaccinations. I inform them about the website szczepienia.info. It’s a credible source of information. The more doctors and nurses talk about vaccinations, the more persons will be inspired to take action” (IDI_17, W, MD).

Some citizens consider their stay in Poland as temporary and therefore they do not take any actions. “Those who stay with us [in Poland] get vaccinated” (IDI_11, W, MD).

“As a country, we should be interested in vaccinating Ukrainians. It seems that both us and them treat vaccinations as a temporary issue. And this is how a gap has emerged” (IDI_4, man [M], MD).

### Preparation, costs and effort

According to the HCPs, Ukrainian mothers face competing priorities such as adjusting to a new country, searching for housing, finding employment, securing access to education for their children (kindergarten, school), learning Polish, and managing other complex mental and physical health needs. These access barriers were often indicated as the main reason behind the low uptake of vaccinations in Ukrainian children. *“*Mothers very rarely ask about vaccinations. On their list of priorities, this topic is very, very low. They have other problems on their minds. Adaptation, confusion. That’s my impression. Vaccinations and infectious diseases are not important to them. Well, they know that diseases exist, but they feel they don’t concern them. Even though doctors talk about the need to get vaccinated and inform patients about a website in the Ukrainian language about vaccinations, where they can read in their own language” (IDI_13, W, N).

Economic barriers and affordability of vaccines are also important in decision making. They can include direct and indirect costs of vaccination, such as cost of travel, and wages lost from time off work. “The prices of combined vaccines also have an impact. When people hear that one vaccination costs several hundred zlotys, this price can be a barrier” (IDI_18, W, MD).

Convenience of access points was a key factor in vaccination decision-making among Ukrainian mothers, particularly those for whom losing a day of work to go to a health center and vaccinate their child may entail significant financial loss. “Vaccinations do not take place every day here. There is shortage in pediatrics; we mainly have elderly doctors. I am afraid that soon there will be no one to qualify for vaccinations. Sometimes vaccinations are only in the morning, and the mother cannot come with the child because she goes to work. This affects accessibility” (IDI_5, W, N).

It is important to improve training and awareness of healthcare workers and other frontline workers about the needs and cultural, religious and social perspectives of refugees and migrants.

### Point of service

Some HCPs felt that the system and services within general practice are not fit for purpose when it comes to meeting the vaccination needs of refugee patients. “We lose track of how many patients disappear from our view; we can’t even estimate it. If they didn’t register at a primary healthcare center, we have no contact with them” (IDI_3, W, MD).

“If we have children from birth, they have a vaccination card. But if the patient is outside the system, if they don’t enter the system, it’s really difficult to track who is missing when you don’t know if they were supposed to be there” (IDI_11, W, MD).

Participants often described going above and beyond their conventional medical role to support refugee patients in various activities, including securing employment, education support, assisting with financial challenges, and helping refugees navigate health services, such as accessing specialist medical care for children with chronic diseases treated in outpatient departments. “At first, on my own initiative, I thought I’d visit various centers. I traveled around, asked questions, and brought various things. I said I was a nurse, that I had been living in Poland for 8 years, and that they could ask me questions, and I would try to explain. I don’t know if it helped, but I saw that these mothers were calmer. And there were so many questions, not silly questions at all. I tried to explain everything to them” (IDI_14, W, N).

“They often ask where to buy glasses for their child. We are not just nurses in our work; we are also people to talk to, who they can discuss health with, but also ask about other things. If we see that someone needs a conversation, we try to meet that need” (IDI_15, W, N).

Completing catch-up immunizations within the recommended timeframe was one of the main challenges, as participants noted. “If we see vaccinations in their booklets, we create individual vaccination schedules and try to catch up. We are keen not to delay this, to vaccinate and immunize as quickly as possible. We talk to parents, we explain” (IDI_3, W, MD).

Everyone agreed that if these barriers were not addressed, future consequences could include outbreaks of VPDs. “This is a very important issue because unfortunately, diseases that we haven’t had are coming back. There are increasing cases of pertussis, tuberculosis, and even measles” (IDI_3, W, MD).

Many participants noted that systems and processes, such as opening hours, recall systems, and administrative processes (e.g. phone bookings), created barriers to health-care utilization for Ukrainian families. “For them, a barrier is that some centers have an extensive patient service with call centers and online bookings, which you have to navigate through” (IDI_17, W, MD).

The standard consultation time for immunization events with refugee children was perceived as too short due to complex health needs, large family sizes, and language barriers. “We, doctors, lack time during medical visits. It would be helpful to extend these visits in order to provide information about vaccinations. Delivering such information as we do for Polish patients requires time, preparation, and also a willingness from Ukrainian patients to listen. Secondly, eliminating the language barrier and providing leaflets in Ukrainian could help” (IDI_2, W, MD).

Patients often presented for visits with medical documentation exclusively in Ukrainian. Sometimes they did not have any vaccination documentation at all, which created problems in determining the vaccination status of the child. “There is no documentation, no vaccination cards. This war has created chaos. Sometimes they are unable to get the documents from Ukraine” (IDI_12, W, N).

“ Understanding the vaccination history involves a lot of our work. We sit down with the doctor and decode the information. We use a translator. Who else is going to do this?” (IDI_5, W, N).

Participants pointed out the phenomenon of missed opportunities to vaccinate. “The topic of vaccinations during infectious visits is not addressed at all. It’s because we have a sense of temporariness. That I help here and now. I know the child is registered at my health center but those files are very thin. Doctors need to be encouraged to engage in vaccination at every opportunity, which I must admit, I don’t do” (IDI_4, M, MD).

Some doctors consider vaccination issues mandatory at every visit, regardless of the patient’s health problem. “During every visit, whether the patient came with an infection or another health issue, we need to inquire about vaccinations. Are the vaccinations appropriate for their age category? Should they have their vaccinations caught up? When I have a first-time patient with a child, I always ask about vaccinations” (IDI_7, W, MD).

Informing about vaccinations is a mission for doctors and nurses participating in this study. “As a doctor, what I can do is try to promote vaccinations during each visit, for the benefit of the patient and the entire population, to prevent the return of dangerous diseases” (IDI_2, W, MD). “Information about vaccinations should come from medical staff. Patients have the right not to be aware of vaccination schedules. They don’t need to know the vaccination calendar. Medical staff should be the ones to bring up this topic” (IDI_17, W, MD).

Communication challenges were recognized as one of the most significant barriers. Sometimes due to language barriers or lack of time, information about vaccination was not transmitted. “Patients are quite specific and difficult. Firstly, there’s a language barrier. They don’t speak Polish well, their English is mediocre, and I don’t know Russian or Ukrainian. I’ve had situations when they got frustrated because I didn’t understand them” (IDI_3,W, MD).

In the absence of an interpreter, many participants used additional communication tools, such as translation apps, visual aids, and sign language. “Somehow you have to manage [...]. Polish and Ukrainian are similar languages. If both sides are willing, they will understand each other, even using gestures. If I didn’t know a word, I looked it up in a translator” (IDI_1, W, N). “We have a Ukrainian doctor who helps us a lot. Sometimes we communicate using gestures, a bit of English, or by drawing on paper. Or we ask the patient to come with someone who can help us communicate” (IDI_13, W, N).

### Experience of care

From the perspective of medical staff, there is no difference between Polish and Ukrainian patients. “They mostly visit the doctors when they have infections. There is also a whole range of pediatric issues. As a nation, they are not healthier than Poles” (IDI_4, M, MD). “However, for me, these are one-time patients. There is some migration, changes in location. We operate more like an emergency service. There are patients who have chronic illnesses. Then I feel more connected to them; they come to us more often, and we know them better” (IDI_4, M, MD).

The medical staff have made efforts to provide the best possible care for war refugees from Ukraine and build a relationship with them. “Patients come back to us, so we have gained some trust from them” (IDI_1, W, N).

“I would really like them to come and get vaccinated. Especially those who are hesitant. Because those who don’t want to come, they don’t come” (IDI_1, W, N).

A need for patient education has been stressed, including informal education, to improve their ability to use the Polish healthcare system, preventive vaccinations, and enhance their satisfaction with received medical care. *“*But when you talk to parents in a non-judgmental, caring atmosphere, they sometimes decide to vaccinate their children. Especially when they understand the consequences of not doing it. Sometimes that really works” (IDI_8, W, MD). “For me, these patients’ mothers are mothers of my children’s friends, neighbors from the same apartment building, and I talk to them about vaccinations in everyday conversations. They speak casually, saying they had the vaccinations and attended appointments. It’s part of their approach to life − if it’s necessary, they do it. It’s not like Polish mothers, who ask a lot of questions about vaccinations and schedules. They don’t pay as much attention to vaccinations” (IDI_15, W, N).

In conversations, the topic of differences in healthcare systems between Ukraine and Poland often emerged. “The healthcare system in Ukraine looks different than that in Poland. They don’t have a comprehensive health assessment by a primary care physician so instead they go to specialists. When we explain our rules, some accept them well while others do not” (IDI_3, W, MD). “I have a feeling that vaccinations are approached less seriously in Ukraine, so here in Poland they are surprised that we are so strict about it. What may be insignificant to them, like a vaccination card, we consider very important and we want the vaccination history recorded. No one will vaccinate a child just based on what someone says, without knowing their vaccination history” (IDI_15, W, N).

The participants pointed out that refugees chose to travel further to access more culturally appropriate practices where staff spoke similar languages or were of similar backgrounds.

The presence of Ukrainian citizens among the medical personnel was a significant advantage. “What works well in the hospital is having two Ukrainian nurses who serve as translators and someone familiar from patient’s country” (IDI_2, W, MD). “In our team, we have doctors from Ukraine and Belarus; they help us, and we refer patients to them. I can call them and discuss cases with them. They also call us for consultations” (IDI_7, W, MD).

### After service

Explanation of the possible side effects of vaccination had a positive effect. Mothers felt reassured after being informed about the symptoms that their child might experience, situations that should be alarming and require medical help and situations when they can manage health issues on their own. “An adverse reaction after vaccination can happen, but it’s important to ask if they know what to do next” (IDI_5, W, N). “Of course, there were questions about whether there would be any complications, whether it would be safe for a child. I spoke directly about possible complications. That was my role to ensure that there wouldn’t be any negative complications, that the child wouldn’t die or end up in hospital. Some mothers didn’t vaccinate their children because someone told them something” (IDI_14, W, N).

It is important to ensure precise information about the next steps, the next vaccination appointment, and to schedule the date. “We have a possibility to register a Ukrainian patient on our list. They become our patient. Then it’s no longer just a one-time patient” (IDI_5, W, N). “We try to meet their needs; we have leaflets in Ukrainian that we had created with the help of Ukrainian doctors. We also have materials from the Ministry of Health and the State Sanitary Inspection, such as the vaccination schedule, which we have translated” (IDI_9, W, N).

Addressing vaccination challenges for mobile Ukrainian migrant populations is crucial, particularly when multiple doses of vaccines are necessary due to the population’s mobility. “They often move from place to place, which makes it difficult to provide care for them at a single health center” (IDI_11, W, MD). “Many patients came to us for their initial vaccinations and then returned to Ukraine, so we don’t know what happened with their vaccinations afterward” (IDI_12, W, N).

It was noted that conventional methods of sending reminder letters and phone calls were ineffective.

## DISCUSSION

The study shows some challenges in delivering vaccinations in Poland to the population of children who are migrants and war refugees from Ukraine, as observed by healthcare workers.

The adopted study framework is helpful in understanding what measures/strategies should be implemented to overcome barriers that child migrants and war refugees encounter at each stage of JHI.

The study results indicate that the healthcare system in Poland is inclusive for Ukrainian migrant and war refugee populations. They receive the same scope of medical care as Polish citizens [19,26]. All migrant children and adolescents without past immunization/vaccine records are offered vaccinations following the Polish vaccination schedules to catch up to the vaccine schedules according to their age group.

The authors’ findings emphasize the critical role of HCPs in providing age-related catch-up immunizations according to align with the Polish National Immunization Schedule, and ensuring optimal protection against VPDs. The experiences of the first-line HCPs could provide valuable insights for improving immunization services at both local and national levels in Poland. It is essential that HCPs are well-informed about migrants’ rights to healthcare services. Thus, HCPs should be provided with continuous training on rights to health care and vaccinations for various profiles of migrants [16,23].

According to the HCPs, the most challenging issues are related to the organizational structure and they include the lack or incomplete medical documentation on vaccination status and previous healthcare provided, especially among children with health burdens such as chronic diseases that require specialist care. In practice, documentation of vaccination status in migrant and war refugee populations is often unavailable or only partially accessible (incomplete information) in host countries, which significantly complicates subsequent medical care and continuity of vaccinations [11,27,28].

There is a lack of an electronic system collecting vaccination data (similar to that dedicated to COVID-19 vaccinations) that would be accessible to both doctors and patients regardless of the country they are in. Archiving data on administered vaccines and its sharing within and among countries has become a crucial challenge to facilitate the completion of a vaccination series and avoid lack or re-vaccination. In the study by Mahimbo et al., both doctors and nurses involved in the vaccination process pointed out that a vaccination registry is an integral tool facilitating vaccination services and it is crucial for assessing needs in this area, especially given the use of vaccinations across different parts of Australia and the high mobility of refugees [29].

In this research, many HCPs expressed concerns about gaps in policies and care for migrants and war refugees, particularly for those who have not registered with a specific family doctor or medical facility and frequently change their place of residence. Such individuals often disappear from the view of healthcare workers responsible for primary and secondary preventive care, including vaccinations and children’s health assessments. Research conducted in Europe has also indicated a lack of guidelines on offering vaccinations to migrants (especially BCG vaccinations) or limited checking of immunization status in refugees and asylum seekers [3,23,30].

At the level of individual migrants and war refugees, HCPs identified multiple factors that either support or impede access to primary care services. These factors included language barriers, transportation, financial barriers, language proficiency, community engagement, and knowledge of the Polish health system.

The opportunity to cooperate with doctors and nurses from Ukraine was highly assessed. Integration of this group in the healthcare system could solve personnel shortages and improve access to medical care for Ukrainian citizens that would be delivered in their native language and cultural context. The language barrier presents itself as a consistent obstacle for communication within the doctor-patient relationship [21,22,27,28].

This study emphasized the importance of maximizing vaccination opportunities. As in the previous studies, HCPs noted that missed vaccination opportunities and delays in vaccinations were often due to administrative barriers, clinic hours, and inefficient internal processes, resulting in a lack of continuity of care. Physicians in Poland and in other countries, still tend to see migrant and war refugee patients more often at problem-focused consultations (e.g., infections) rather than in the framework of preventive care (e.g., screenings). This stresses the need to use every meeting with a patient, even during their illness, to increase the vaccine uptake [5,27].

The unique observations of HCPs presented in this study provided further insight into the systemic challenges faced by healthcare workers in delivering medical care to migrants, including vaccination services.

The study revealed that the success of healthcare workers in achieving high vaccination coverage among the Ukrainian patients is determined by the healthcare system. There are numerous factors in the healthcare system and broader socio-political system that can either strengthen or mitigate challenges faced by both HCPs and patients [31].

Frontline healthcare workers who interact directly with patients require systemic support, including guidelines, educational materials for patients, and organizational changes (such as extended appointment slots). It is necessary to provide training opportunities for HCPs to improve their awareness of the catch-up needs of refugees across all age groups. Resources such as online immunization calculators, refugee specific guidelines and e-learning could potentially equip HCPs with the relevant skills and knowledge and ultimately make implementation of catch-up vaccines for this group easier [14,27,29].

Continuous training and updating are essential for strengthening the health system and other sectors involved in vaccination [5,16].

The lack of adequate knowledge and skills in providing culturally competent care to refugees has often been presented in the literature [22,31].

A range of resources have been developed to facilitate the delivery of comprehensive healthcare services to refugees from Ukraine. These include informational leaflets outlining the process of navigating the Polish healthcare system and websites featuring materials covering mandatory and recommended vaccinations [11]. The Supreme Chamber of Physicians and the Centre of Postgraduate Medical Education addressed, i.a., language and cultural barriers by providing training for HCPs and posting resources in Polish and Ukrainian on their websites. This initiative aims to enhance communication between healthcare workers and Ukrainian patients [32,33].

The authors’ findings underline the need to review barriers faced by healthcare workers and provide them with training on migrant health and medical care. The more awareness practitioners have about their patients’ cultural backgrounds, the higher the quality of care they can provide [11,14,28].

### Strengths and limitations

Strengths of this study include a qualitative approach and using the JHI framework, which allowed us to gain a deeper understanding of the topic of vaccination among Ukrainian children from the HCPs’ perspectives. The nurses and physicians were recruited from both primary and tertiary care settings and the public and private sectors, which allowed us to gather different points of view and opinions. Qualitative studies have limitations. The results reflect the opinions of a limited number of HCPs and should not be generalized. The study’s findings are based on 4 specific locations and may not be representative of the entire country or the full range of factors impacting vaccination services for Ukrainian children in other host countries. Additionally, the interviews were conducted online, which could have potentially hindered effective communication.

Despite these limitations, the study results could inform the application of behavior change interventions among HCPs, patients, and parents/caregivers to facilitate optimal preventive care services and increase vaccination rates among Ukrainian children in the host country.

## CONCLUSIONS

Overcoming barriers related to vaccinations for migrant and war refugee populations requires a comprehensive approach, including building HCPs’ knowledge about migrants’ rights to healthcare services and vaccinations, improving communication between patients and HCPs, enhancing vaccine literacy, implementing informational campaigns to build trust in vaccinations and achieving vaccination coverage through flexible, tailored systemic solutions. The conducted study provides new insights into both strengths and weaknesses of vaccination programs for Ukrainian children in Poland, and it also offers a deeper understanding of the barriers and drivers to vaccination. This understanding can potentially lead to more effective interventions. Identified barriers and challenges do not fundamentally differ from those observed in other countries, e.g. gaps in vaccination coverage.

Since the war in Ukraine continues, migrant and war refugee populations cannot safely return to their home country. Therefore, the vaccination of these populations has become a key priority in host countries.
